# *Akkermansia* and Microbial Degradation of Mucus in Cats and Dogs: Implications to the Growing Worldwide Epidemic of Pet Obesity

**DOI:** 10.3390/vetsci7020044

**Published:** 2020-04-15

**Authors:** Jose F. Garcia-Mazcorro, Yasushi Minamoto, Jorge R. Kawas, Jan S. Suchodolski, Willem M. de Vos

**Affiliations:** 1 Research and Development, MNA de Mexico, San Nicolas de los Garza, Nuevo Leon 66477, Mexico; 2 Animal Emergency & Specialty, Kirkland, WA 98034, USA; yasushi.minamoto@gmail.com; 3 Faculty of Agronomy, Universidad Autonoma de Nuevo Leon, General Escobedo, Nuevo Leon 66050, Mexico; jorge.kawas@mnademexico.com; 4 Gastrointestinal Laboratory, Department of Small Animal Clinical Sciences, College of Veterinary Medicine and Biomedical Sciences, Texas A&M University, College Station, TX 77843-4474, USA; jsuchodolski@cvm.tamu.edu; 5 Laboratory of Microbiology, Wageningen University, 6708 WE Wageningen, The Netherlands; willem.devos@wur.nl; 6 Human Microbiome Research Program, Faculty of Medicine, University of Helsinki, P.O. Box 63, 00014 Helsinki, Finland

**Keywords:** feline obesity, canine obesity, mucus degradation, companion animals

## Abstract

*Akkermansia muciniphila* is a mucin-degrading bacterium that has shown the potential to provide anti-inflammatory and anti-obesity effects in mouse and man. We here focus on companion animals, specifically cats and dogs, and evaluate the microbial degradation of mucus and its health impact in the context of the worldwide epidemic of pet obesity. A literature survey revealed that the two presently known *Akkermansia* spp., *A. muciniphila* and *A. glycaniphila*, as well as other members of the phylum of Verrucomicrobia seem to be neither very prevalent nor abundant in the digestive tract of cats and dog. While this may be due to methodological aspects, it suggests that bacteria related to *Akkermansia* are not the major mucus degraders in these pets and hence other mucus-utilizing taxa may deserve attention. Hence, we will discuss the potential of these endogenous mucus utilizers and dietary interventions to boost these as well as the use of *Akkermansia* spp. related bacteria or their components as strategies to target feline and canine obesity.

## 1. Introduction

Obesity is a complex disease that is closely related to host genetics, environmental factors, and the microorganisms inhabiting the digestive tract (gut microbiota). According to the World Health Organization, globally, there are more than 1 billion overweight adults, and at least 300 million of these clinically obese [1]. Obesity is associated with additional medical problems, such as cardiovascular, musculoskeletal, and psychological disorders, diabetes, and an overall lower quality of life.

According to the American Veterinary Medical Association (AVMA), there are approximately 76 million dogs and 58 million cats in the US, and approximately 60% of these animals are overweight or obese [2]. Obesity is one of the most common nutritional imbalances in companion animals [3,4] and carries similar medical problems as in people. Traditional therapies to treat obese cats and dogs include exercise as well as changes in diet and dietary patterns [5]. More recently, the use of beneficial microorganisms has been considered as a feasible alternative solution in obese people [6]. However, the development of effective bacterial therapies with weight-loss applications in obese cats and dogs has shown to be challenging [7], in part due to the wide inter-individual variation in the gut microbiota of these animal species. 

*Akkermansia muciniphila* is a mucin-degrading bacterium that has shown the potential to provide anti-inflammatory and anti-obesity effects in mouse and man by reducing insulin resistance, glucose intolerance, and gut permeability. We here focus on microbial degradation of intestinal mucus and its health impact in the context of the worldwide epidemic of feline and canine obesity.

## 2. The Gut Microbiota

From birth, and perhaps even before that, the digestive tract is inhabited by a complex mixture of microorganisms, including bacteriophages and other viruses. The density of microbial life varies widely throughout the gut. The stomach and the small intestine contain small numbers of bacteria (<1 × 10^4^ bacteria/mL, mostly attached to the mucosal layer with little luminal microbes), while the large intestine is much more densely populated (>1 × 10^11^ microbial cells/g of intestinal content) [8]. Despite the high microbial density, in normal circumstances, the gut microbiota lives in equilibrium with the immune system from the host [9]. However, in the event of physiological or physical alterations, for example when the mucosal barrier is compromised, the host–microbiota relationship is no longer in balance, and a process of inflammation may develop [10,11,12]. This is an area of great interest particularly for clinicians because the manipulation of the host–microbiota milieu inside the gut, such as bacteria marketed as probiotics, can lead to better strategies to maintain and restore health.

In human beings, it has been shown that each individual harbors a highly individualized microbiota inside the gut [13], a finding that has been reproduced at large scale that showed even personalization at the strain level [14]. While a core microbiome can be identified, the microbial signature in each individual may be better considered as an ‘evolving fingerprint’ since the gut microbiota also shows pronounced temporal variability across months, weeks, and even days [15,16]. This uniqueness of the gut microbiota is also true for pet animal species as shown by a growing number of studies in cats and dogs (see “The feline and canine gut microbiota” below). While an evolving individualized microbiota profile may be difficult to visualize, this makes sense when considering the individuality and evolution of microorganisms and host–microbe interactions [17].

The gut microbiota, particularly that in the large intestine, is closely related to energy homeostasis, fat metabolism, and obesity. One seminal study showed that colonization of adult germ-free mice with a distal gut microbiota harvested from conventionally raised mice produced a remarkable increase in body fat within two weeks, despite an associated decrease in food consumption [18]. On the other hand, certain diets in mice can induce obesity and this phenomenon is linked to marked alterations in the distal gut microbiome, although specific microbial manipulations may limit weight gain [19,20]. While these and other studies have shed light into the complex host–microbiota relation in obesity, a causal relationship has been found difficult to establish [21].

The gut microbiota is composed of hundreds of different types of microbes with wildly different ecophysiologies [22]. Within this complex and unique assortment of microorganisms, some microbes have apparently evolved to live in the mucus, a heavily glycosylated protein that is continuously produced to cover and protect the inner epithelial layer of the digestive tract. Some of these mucus-degraders have specialized in surviving on host-produced mucus rather than diet-derived compounds that feed most of the colonic bacteria. It has been even suggested that bacteria that live in the mucosa do not compete with the microbiota present in the lumen and, therefore, do not depend on dietary nutrients deriving from host food consumption [23,24]. This is a very important corollary that will be discussed again in the last section of this article (see “Implications of microbial mucus degradation for health in cats and dogs” below).

## 3. Gastrointestinal (GI) Mucus

The digestive tract of humans and other animals is a muscular tube covered on the inside by epithelium containing mucus-secreting goblet cells [25,26,27]. This mucus has important protective, lubricative, and immune functions that are relevant to preserve health [28,29,30,31]. Studies in laboratory animals have shown that the stomach and the small intestine are covered by one layer of mucus (~100 μm), while the large intestine has a two-layered mucus (~150 μm) that differs in biochemical composition, structure, and relationship with the microorganisms inhabiting the gut [32,33,34,35,36].

Biochemical studies showed that the mucus layer is composed of two layers, a sterile inner layer that protects the enterocytes and an outer layer that is colonized and provides nutrients and attachment sites for the intestinal bacteria [37]. In this context, it should be noted that mucus production in the animal intestinal tract is high and may account for approximately half of the carbon present in the colon and hence is an important substrate for bacteria that are fed directly by the host [38]. In addition, it was found that development in the colon of mice takes several weeks, and that is a phenomenon with a close dependence on the presence and metabolic activities of different groups of microorganisms [39]. These findings are relevant, especially in a context of GI diseases that involve destruction of mucus and mucosal epithelium, for example inflammatory bowel disease (IBD, [40,41]). The unbalanced destruction of mucus has several negative consequences for the host, including a diminished defense of enterocytes, an increased presence of mucus-associated bacteria in the lumen, and more nutrients to luminal bacteria that usually do not feed on these substrates. These consequences may lead to a more unbalanced state during GI disease, and it has been proposed that this leads to biofilms that are often observed in patients suffering from IBD or colorectal cancer [11,42,43].

The production and release of mucus is a phenomenon affected primarily by anatomical location, host genetics, and pathogenic infections, as well as the gut microbiota and their metabolites (e.g., short-chain fatty acids (SCFAs), acetate, propionate, and butyrate). One early study showed that SCFAs did not influence the number of cells staining for mucin in the rat colon, but solutions of low osmolality (i.e., with a low number of dissolved particles) caused a considerable release of mucin from goblet cells [44]. Other, more recent, studies have shown that certain dietary ingredients can lead to enhanced production of SCFA or lactic acid, which in turn may have stimulating effects on mucus production and affect epithelial barrier integrity in mouse and man [45,46]. On the other hand, each animal species is unique in terms of dietary and physiological characteristics, and therefore, it is expected that each species also possesses a distinctive mechanism of mucus production as well as different mucus structure and biochemical composition.

Several studies have investigated the physicochemical and antigenic characteristics of GI mucus in health and disease in humans [47,48,49,50], while others have focused on mice [28,51,52,53]. To our knowledge, there have been no comparisons of mucus physicochemical properties among animal species, in part due to the inherent complications of analyzing mucus separately of intestinal contents. An early study used a radioimmunoassay for the quantitative measurement of human goblet cell mucin and showed that human goblet cell mucin cross-reacted with dog, monkey, and rabbit mucins, but not with mucins of rat, pig, toad, and oyster [54].

### A. muciniphila and Microbial Degradation of Mucus

The gut microbiota affects the whole-body metabolism by affecting the energy balance and gut permeability, particularly in the large intestine. *A. muciniphila* was first isolated in 2004 at Wageningen University from a healthy subject as an abundant specialist growing on animal and human mucus [55]. Later on, it was demonstrated that *A. muciniphila* cells are related to energy storage and whole-body metabolism by improving insulin sensitivity, reducing insulinemia and plasma total cholesterol, increasing the intestinal levels of endocannabinoids that control inflammation, the gut barrier function, and gut peptide secretion in mouse and man [20,56]. It remains to be seen whether these properties could also be linked to other mucus-degrading bacteria. However, these findings help clarifying, at least partly, the relationship between microbial degradation of mucus and obesity.

Presently, only two recognized species of the genus *Akkermansia* have been described. *A. muciniphila*, found in man and many other species, and *A. glycaniphila* isolated from a Burmese python [57,58]. A recent genomic study of 70 human strains suggested the presence of four species-level groups, although the 16S rRNA sequences of all isolates showed more than 97% identity [59,60]. Of interest, two of these new isolates could produce vitamin B12 and contained corrin ring biosynthesis-associated genes, similar to *A. glycaniphila* [59]. Several review papers have summarized the state-of-the-art knowledge on mucin–bacterial interactions and *A. muciniphila* [24,58,61,62,63]. Hence, we focused here on particular aspects of this bacterium that have not been discussed and are relevant for this work. Interestingly, *A. muciniphila* has been investigated for its effect on systemic markers of GI permeability and epithelial damage following antimicrobial administration in dogs [64].

*Verrucomicrobium spinosum* was the first isolated organism of a new phylum that is now known as the Verrucomicrobia and was described in 1987 [65]. This bacterium was given such name because of the presence of bundles of fimbriae originating from the tips of the prosthecae. Different types of microbes are currently catalogued within the Verrucomicrobia, including some without the fimbriae in *V. spinosum*. The phylogenetic relationship of Verrucomicrobia with other bacterial groups is constantly evolving. For example, it has been suggested that Verrucomicrobia, Planctomycetes, and other groups form a monophyletic group referred to as the PVC superphylum [66].

It has been suggested that *Akkermansia*-like organisms are universally distributed in the intestines of the animal kingdom [24]. However, it was also observed that *Akkermansia* spp. were not detected in all taxa within the order of the Mammalia, which at that moment was explained by the possibility that *Akkermansia* sequences might have been overlooked because the sequence depth was limited. Others have also suggested a global distribution of *A. muciniphila* in the mammalian gut microbiota but have only used data from feces of humans, mice, and pigs [67]. In an early massive study of the microbiota in mammals, it was shown that most Verrucomicrobia 16S rRNA sequences belonged to *Akkermansia* but also to the Class Subdivision 5 (1054 sequences in the Ribosomal Database Project, RDP) [68]. Moreover, following the characterization of *A. muciniphila* type strain at the genome level and using this in human metagenomic libraries, it was found that there may be new and uncultured *Akkermansia* species, since metagenomic sequences with 84–88% average nucleotide identity were detected [69]. This study also indicated that there could be at least eight different representative species within the *Akkermansia* genus based on the 16S rRNA gene sequences when a cut off of 98% identity was used and suggested that some individuals may be colonized by different species of *Akkermansia* [69]. The latter makes sense when considering the individuality in bacteria [17] and was later confirmed experimentally [70,71]. It is also important to remember that identical 16S rRNA gene sequences have been found in microorganisms with highly divergent ecophysiologies [72], a highly relevant example being the *Escherichia coli* K12, an innocent gut isolate, and *E. coli* O157, responsible for many disease outbreaks [73]. This indicates that the metabolic potential cannot always be deduced from the taxonomic position of an *Akkermansia*-like isolate. However, microbial taxonomy will always be based on cultured and deposited representatives and is still a polyphasic approach including the use of phenotypic, chemotaxonomic, and genotypic data [74].

**Figure 1:**
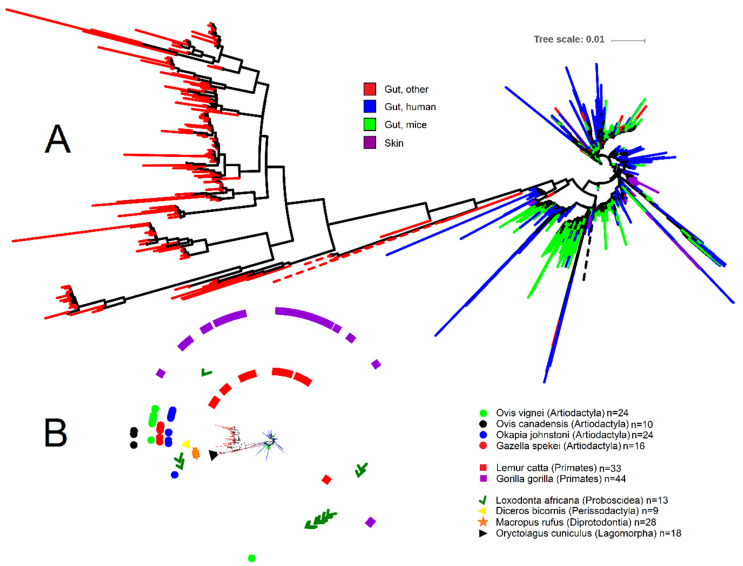
Phylogenetic portrayal of all 1872 16S rRNA gene sequences classified as *Akkermansia* from the Ribosomal Database Project. (**A**) Circular phylogenetic tree showing the relationship of 16S rRNA gene sequences (note that most sequences belonging to the category of other animal species clustered together—“Gut, other” in red; other sequences from this category clustered with human and mice samples but are hard to visualize due to sequence overlap). The tree was generated using FastTree [76] in QIIME [77] 1.8. (**B**) Miniaturized circular tree from Figure 1A showing symbols for each animal species from the study by Ley et al. [68]. *Akkermansia* sequences clustered closely together for several animal species (*Oryctolagus cuniculus*, *Diceros bicornis*, and all animal species within the order Artiodactyla), suggesting host specialization. The most dispersed *Akkermansia* sequences belonged to the order of Primates (*Lemur catta* and *Gorilla gorilla*) and Proboscidea (*Loxodonta africana*). Note that a total of eight sequences from the order Perissodactyla (three sequences from *Equus equus*, one sequence from *Equus grevyi*, four sequences from *Equus asinus*), 17 sequences from the order Artiodactyla (three sequences from *Budorcas taxicolor*, four sequences from *Sus cebifrons*, six sequences from *Babyrousa babyrussa*, one sequence from *Ovis ammon*, one sequence from *Giraffa camelopardalis* reticulata, and two sequences from *Antidorcas marsupialis*), four sequences from the order Carnivora (*Acinonyx jubatus*), one sequence from the order Chiroptera (*Pteropus giganteus*), and two sequences from the order Rodentia (*Callosciurus prevosti*) are not shown for sake of visual clarity. Tree scale refers to sequence dissimilarity (the longer the scale the bigger the dissimilarity).

To help shed light onto the nature and distribution of *Akkermansia* spp., we depicted the phylogenetic relationship of all 1872 *Akkermansia*-like 16S rRNA gene sequences (>1200 nucleotides, common gaps removed) retrieved from the RDP database (Figure 1; analysis done by December 2019) using iTOL [75]. *Akkermansia* is one of the best represented genera within the Verrucomicrobia in RDP, accounting for ~17% of all 10,731 16S rRNA sequences of Verrucomicrobia. From the *Akkermansia*-like sequences, a subset of 250 sequences (~13% of all *Akkermansia* sequences in RDP) belong to the study of fecal microbial communities from 60 mammalian species from 13 taxonomic orders [68]. Note that RDP contains a total of 16,763 16S rRNA gene sequences from this study, most of which belong to Firmicutes (10,697 sequences), Bacteroidetes (2636 sequences), and Proteobacteria (1579 sequences) (these three taxa account for 89% of all sequences from Ley et al. [68]). Interestingly, 219 sequences from this study also belong to Verrucomicrobia but were catalogued within the Class Subdivision 5, including taxa that we did not discuss here but may deserve attention in future studies.

The analysis of *Akkermansia*-like16S rRNA gene sequences from RDP offered several important insights. First, the majority of *Akkermansia*-like 16S rRNA sequences belonging to non-human and non-mice gut contents clustered together (bootstrap support 0.862); of these, most belonged to the study of zoo animals (Figure 1). Second, this cluster showed a much stronger sequence dissimilarity compared to the rest of the sequences, as evidenced by the longer tree scale (Figure 1). Third, *Akkermansia*-like 16S rRNA sequences were present in only nine out of the 13 orders sampled by Ley et al. [68] (Figure 1). While this may suggest that *Akkermansia*-like bacteria is not universally distributed across the mammalian kingdom, as indicated above, this may also be due to limited sequence depth or absence in the animal species sampled. Fourth, *Akkermansia*-like 16S rRNA sequences from a given animal species sometimes clustered together (for example for the European rabbit *Oryctolagus cuniculus* and Black rhinoceros *Diceros bicornis*), thus suggesting host speciation even though different individuals from each animal species were sampled. However, one should keep in mind that the vast majority of samples derived from zoo animals that may have been housed together or exposed to the human species. This can be exemplified from the genomes of *A. muciniphila* isolated from laboratory mice that are almost identical to that of the *A. muciniphila* human type strain. On the other hand, several *Akkermansia* 16S rRNA sequences from other animal species (e.g., samples from Primates) were more dispersed in the tree and clustered together with human and mice sequences (Figure 1), thus suggesting that host-speciation is different among animal species. Finally, several sequences from two different studies [78,79] were obtained from skin, thus confirming the presence of this organism in this environment [80]. However, several of these sequences derived from breasts of women that may have been lactating and it is known that *A. muciniphila* is present in breast milk and can grow well on human milk oligosaccharides [71,81]. Overall, these insights suggest either a) that different animal species are colonized by different types of *Akkermansia*-like bacteria, or b) that the same types of *Akkermansia*-like bacteria colonize different hosts. This latter hypothesis has also been suggested by others [24], but it is very well possible that both options are feasible and not necessarily exclude each other.

Lastly, we and others suggest that 16S rRNA sequences from *Akkermansia*-like and other low abundant bacterial groups may not be detected even from massive sequencing surveys due to inadequate sequence depth (see “The feline and canine gut microbiota” below). Therefore, it is important to briefly discuss the numbers of *A. muciniphila*. Relative abundance of *A. muciniphila* in the colon of healthy humans is approximately 3% [63] (the minimum reported is often 0.1%, and the maximum reported is up to 85% [69,82]). Now, relative percentages of 16S rRNA gene reads cannot be accurately translated into cell numbers, but in an environment of 1 × 10^11^ bacteria/g of contents in the large intestine, these percentages would imply approximately 1 × 10^8^ to 3 × 10^9^ bacteria/g of contents. These hypothetical numbers of cells based on sequence reads agree with other studies that have looked at the actual numbers of *A. muciniphila*. Collado et al. [83] showed 1 × 10^8^ cells/g in feces, and Derrien et al. [84] showed that *A. muciniphila* accounted for more than 1% of the total fecal cells, which in an environment of 1 × 10^11^ bacteria/g of contents would represent 1 × 10^9^ cells. Another study showed that one single dose of 10^9^ *A. muciniphila* to germ-free mice yielded 1.9 × 10^8^ cells/g of ileal contents, 3.1 × 10^10^ cells/g cecal content, and 1.7 × 10^9^ cells/g colonic content [23]. Another more recent study has also shown that viable *A. muciniphila* cells can be recovered from mice cecal and colon contents in a concentration of up to 1 × 10^10^ cells/g [70]. Overall, these studies agree with a possible concentration of 1 × 10^8^–1 × 10^10^ in large intestinal contents. Now, in a regular sequencing survey using, for example, the Illumina MiSeq platform, an approximate 30,000 16S rRNA reads are analyzed, and 0.1–4% represents only 30–120 sequences. These estimations, albeit imprecise, strongly suggest that in the case that *Akkermansia* or other microbes are present with an abundance lower than 0.1% in sequencing surveys of 30,000 sequences/sample, we should not overlook taxa with less than 100 sequences, as done by others [85]. However, an important point to make here is that the presence of an *Akkermansia*-like 16S rRNA sequence in DNA isolated from a given sample does not necessarily supports the presence of *Akkermansia*-like bacteria in the habitat where that samples are taken from. Many artefacts related to cross-sample contaminations have been reported, notably with the use of sensitive PCR methods as well as 454 and Illumina sequencing approaches. Hence, relying on a limited number of sequences has a risk, and with the present new platforms, such as the Illumina NovaSeq, it may be possible to have very deep analyses of fecal and other samples, amounting to more than millions of reads.

## 4. The Feline and Canine Gut Microbiota

Similar to humans and other mammals, cats and dogs harbor a complex assemblage of different types of microorganisms inside their digestive tracts. Also, each section of the GI tract has different physiological conditions, including complex defense mechanisms [86] that in turn help develop different microbial communities [87,88,89,90,91].

As indicated above, the development of microbial therapies with weight-loss applications in obese cats and dogs is challenging [7], in part due to the little we know about microbes and obesity [92,93] in these animal species and the wide inter-individual variation in the gut microbiome. This variation in the gut microbiome in pets is associated with the passing of time and aging [94,95,96,97,98], environmental factors, including diet [99], microbes in surrounding people [100], breed and other host genetics factors [101,102], clinical and subclinical conditions [91,93,103], gender [104], and behavior [105]. Here, we discuss the literature on feline and canine gut microbiota with regards to Verrucomicrobia- and *Akkermansia*-like 16S rRNA sequences (Table 1).

### 4.1. Oral Cavity

Verrucomicrobia has not been detected in the oral microbiota of cats [115,116,118]. An early molecular description of the canine oral microbiota (5958 full-length 16S rRNA gene sequences) did not find any sequence belonging to Verrucomicrobia in this environment [112]. Other studies of the canine oral microbiota have also failed to show the presence of Verrucomicrobia [147,148]. Similarly, Verrucomicrobia have not been detected in the human oral cavity [58,149,150,151,152], although a single report describes the presence of *Akkermansia*-like sequences in the oral cavity of a choledocholithiasis patient [153]. The Human Oral Microbiome Database [154,155] also does not contain any information about Verrucomicrobia. These studies strongly suggest that *Akkermansia* is absent from the healthy oral cavity of humans, cats, and dogs, something that could be explained by its strict anaerobic nature as described by Derrien et al. [55] although the type strain can tolerate and use small amounts of oxygen [156]. However, several anaerobes have been described and isolated from the human oral microbiota, but these often include pathogens that are colonizing deep and anerobic pockets or are in specific biofilms [43,157]. However, recent data indicate that *A. muciniphila* can be used to prevent some pathogens like the anaerobic *Poryphyromonas gingivalis* to produce gingivitis and cause bone loss in mouse models [158].

### 4.2. Stomach

The studies of the feline gastric microbiota have focused on specific bacteria such as Helicobacter [159] and no Verrucomicrobia was detected in the stomach of healthy dogs using high-throughput 16S rRNA sequencing [104]. Similarly, Verrucomicrobia has not been detected in some studies of the human gastric microbiota [160,161]. This makes sense at least when considering that the original A. muciniphila strain MucT did not grow below pH 5.5 or above pH 8 [55] while A. glycaniphila grew best between pH 5.0 and pH 7.5 [57]. However, it remains possible that other and novel Akkermansia species may grow at more extreme pH values.

### 4.3. Intestinal Tract

Verrucomicrobia has been detected in very low abundance (0.1%, [110]) or not at all [41,108] in the canine duodenum. Moreover, an analysis of almost all sections of the digestive tract of cats [106] and dogs [107] did not detect Verrucomicrobia. More recent studies of the small and large mucosal microbiota have also failed to detect Verrucomicrobia in dogs [91] or not attempted to look for this group at all [162]. These and other studies (Table 1) strongly suggest that *Akkermansia* has a very low prevalence and is not an abundant member of the microbiota in the small intestine of cats and dogs. Possible explanations for this include the fact that the mucosal layer in the small intestine is thinner and less dense. This fact and other biochemical conditions (e.g., presence of bile) may limit microbial life in this section of the GI tract. An alternative explanation can be the fact that dogs, and to some extent also cats, are highly inbred and their microbiota may be dependent on the breeders. It has been shown that laboratory mice derived from different suppliers have a greatly variable microbiome, including a highly varying level of *Akkermansia* spp. [163].

A great number of studies have analyzed the fecal microbiota in cats and dogs, and most have failed to detect Verrucomicrobia. Exceptions include the study by Handl et al. [111] where *Akkermansia* was not detected in any fecal sample from 12 healthy pet dogs and in only 1 fecal sample from 12 healthy pet cats, albeit in very low proportion (0.01%) (in relation to the numbers discussed above, it is interesting that this study used 454-pyrosequencing and generated only ~5000 sequences per sample, and 0.01% of these equals approximately one sequence only). In another study comparing the fecal microbiota between lean (n = 21) and obese (n = 22) dogs (also using low-throughput pyrosequencing), Verrucomicrobia were detected in only one lean dog and in very low abundance (<0.01% [92]). A recent study of household pet cats (n = 46) and dogs (n = 192) showed that the abundance of fecal Verrucomicrobia was 0.11% in cats and 0.02% in dogs [138]. Similarly, some studies of the human fecal microbiota have not detected this group either [164] and others have detected it in high levels in only a few sections of the digestive tract [165], suggesting that either *Akkermansia* is not that universally distributed or that its abundance in some individuals is simply undetectable. One more recent study used a FISH probe developed previously [84] to look for the first time into the abundance of *Akkermansia* in colonic mucosa of healthy dogs and dogs with chronic enteropathy [136]. Interestingly, the abundance of *Akkermansia* was similar to other microbes such as *Faecalibacterium*, and it was statistically higher in healthy dogs. This topic indeed deserves more attention with regards to prevalence and abundance, for instance one study of non-human primates showed that Verrucomicrobia was relatively highly abundant in feces (0.3–2.6%) but common to all nine subjects from three different primate species [166].

### 4.4. Verrucomicrobia in Other Anatomical Areas

While *A. muciniphila* is often regarded as part of the human colonic mucus-associated microbiota, recently, the intriguing possibility was discussed of having *Akkermansia* in other anatomical areas such as the pancreas and gallbladder [58]. However, only few studies have analyzed bacteria and other microbes in these organs in cats or dogs [167]. To our knowledge, *Akkermansia* and other members of Verrucomicrobia have not been searched or detected in these sites. Moreover, Verrucomicrobia has not been detected in the urinary microbiome of healthy dogs [168], the microbiota in bronchoalveolar fluid and blood of healthy cats [169], or the upper and lower airway microbiota of healthy dogs [170]. Verrucomicrobia have not been detected in the skin microbiome of healthy and allergic cats and dogs [171,172,173], and this deserves special attention because Verrucomicrobia has been found in human skin [80].

## 5. Why is *Akkermansia* So Rare in the Digestive Tract of Cats and Dogs?

The presence or absence of certain types of microorganisms in specific ecological niches is intriguing and has important implications in several fields of science [174,175]. In the case of the GI tract of humans and animals, this topic becomes especially relevant in a context of health and disease. On one extreme, we have the abundant and prevalent microbes, sometimes referred to as the core microbiota, a continuously revisited concept [176]. Several efforts have been directed to identify a possible relationship between this core microbiota with intestinal symptoms of disease [15] or metabolic diseases such as obesity [177]. In this context of core microbiota, it is interesting that *Akkermansia* has been shown to co-occur with two of the three main enterotypes in humans as previously described based on metagenomic information [178].

On the other extreme, we have the rare and less prevalent microbes, which have been detected only in a few individuals and in very low abundance or not at all. Unfortunately, we know little about the contribution of these low abundant groups to overall gut homeostasis [179]; in fact, some researchers suggest and promote the removal of low abundant microbes from analysis in order to detect “true” phylotypes [180]. This is not a trivial topic, at least in other environments such as soil it has been shown that the low-abundance bacteria play a fundamental biological role [181] and may, in fact, be keystone species regulating the function of different microbial environments, including host-associated microbiomes [182].

With few exemptions, the literature search found no evidence to believe that *Akkermansia* is present in high prevalence or abundance in any portion of the digestive tract of cats and dogs. Therefore, we suggest that this bacterium (and in fact the whole Verrucomicrobia) is not part of the core feline or canine gut microbiota. But why not? One technical reason may relate to sequencing depth and this has also been suggested by others [24]; in fact, based on our calculations described above, a relative abundance lower than 0.1% would be difficult to detect when having up to 30,000 sequences per sample. In our own experience, we have detected high levels (up to 5%) of *Akkermansia* in fecal samples from mice [183] using the same sequencing depth (minimum 60,000 sequences per sample) as some of our more recent studies in cats and dogs. Another reason may be related to the use of 16S rRNA primers and probes. Members of Verrucomicrobia are deeply rooted and some new species may not be detected with the canonical universal primers. This is particularly important when using other techniques such as FISH because the 16S rRNA sequence of *A. muciniphila* contains two mismatches with the traditional universal bacterial probe EUB 338 [84]. Another reason for not having *Akkermansia* may be related to the reference sequence database used to select OTUs (for example, the one from GreenGenes). However, even the earliest version of this database (August 2012) already contained sequences belonging to *Akkermansia* spp. and other taxa within the Verrucomicrobia. Another possibility is that the *Akkermansia*-like sequences found in the studies of the cat and dog microbiota were very divergent compared to the reference *A. muciniphila* or *A. glycaniphila* sequences and hence did not match with the reference sequences (the fate of these sequences will depend on the OTU clustering method, some methods, such as the pick_closed_reference_otus.py in QIIME1, discard the most divergent sequences to be able to detect only “true” phylotypes). This is indeed a possibility especially when considering, for example, the existence of non-*Akkermansia*-like sequences in databases such as the Class Subdivision 5 in RDP from the same samples published previously [68]. It should also be noted that some studies have shown a high abundance of Verrucomicrobia (~4%) in feces of horses [184], while it was shown that *Akkermansia* spp. were present in only a few samples of horses or other members of the order Perissodactyla [68]. Indeed, as indicated previously, the nature and divergence of 16S rRNA genes of *Akkermansia*-related organisms is worth investigating [24].

If we assume the obligatory existence of bacteria that degrade mucin and other host-compounds, then other bacteria must be playing the function of *Akkermansia* spp. in cats and dogs. In fact, using an elegant approach, it was shown that *Bacteroides acidifaciens* was also an important host-compound forager in vivo, and *A. muciniphila* and *B. acidifaciens* received more attention in this study only because they were the more abundant ones (other microorganisms such as members of Ruminococcaceae, Lactobacillaceae, Enterococcaceae, and Mucispirillum were also enriched in host-secreted proteins) [185]. Indeed, mucin degradation is phylogenetically widespread [186], and actually, the outer mucus layer contains bacteria without specialized mucolytic capabilities [187]. This has been elegantly explained by the fact that the structural heterogeneity of the substrates and the associated enzymatic diversity required for complete mucus degradation needs an assortment of many different microorganisms [188]. In dogs, different microorganisms have been shown in mucosal samples of healthy dogs (e.g., Bacteroidaceae, Prevotellaceae, Clostridiales, *Faecalibacterium*, and the Fusobacteria phylum [41,136], and these groups may in fact represent some of the native mucus degraders in these animal species.

## 6. Implications of Microbial Mucus Degradation for Health in Cats and Dogs

Microbial mucus degradation is an important phenomenon to consider in feline and canine gut health. Several lines of research can emerge from not having *Akkermansia* spp. in cats and dogs in high prevalence or abundance.

More research is needed to investigate the type of mucins produced and secreted by the GI tract of cats and dogs. For example, more than 20 genes encoding mucins have been identified in humans, and different mucins have been found in the different sections, including the stomach [26]. Further, more research is necessary to reveal the identities and quantities of the most predominant mucus-degraders as well as the extent of host-compound foraging in cats and dogs. For this end, one can use a similarly elegant but complex approach as used previously with high-resolution secondary ion mass spectrometry (NanoSIMS) combined with fluorescence in situ hybridization to determine the isotope content of individual cells hybridized with specific phylogenetic probes [185]. Note that whether *A. muciniphila* is also a major host-compound forager in humans has not been proven in such studies that use labeled amino acids.

Obesity is a growing problem in cats and dogs and several lines of research point out a potential role of the gut microbiota in this disorder [189,190,191,192,193]; therefore, several efforts have been directed to help these patients but mostly centered around dietary therapies [3]. *A. muciniphila* is currently considered a beneficial microbe that could help in the treatment of obesity, diabetes, and associated disorders in humans when administered orally, either alive or even pasteurized [56,194,195]. Given the increased prevalence of obesity in cats and dogs, the question remains as to whether *Akkermansia* spp. could also be considered as an option for a therapy for obesity in these animal species. Note that probiotics do not necessarily need to be isolated from the same animal species, or even from the same environment, in order to survive and colonize the intestinal tract temporarily [196]. Therefore, even if *Akkermansia* spp. are not considered native to the feline and canine gut, both *A. muciniphila* or *A. glycaniphila* could nonetheless be considered for these animal species. Another option to consider is the use of a purified membrane protein from *A. muciniphila* that interacts with Toll-like receptor (TLR) 2 and recapitulates the beneficial effects of the bacterium [195]. This correlation with TLR2 is important, because of its involvement in maintaining intestinal homeostasis, bacterial recognition, and host metabolism [197]. The signaling to TLR2 is also particularly relevant in cases of IBD in veterinary medicine [198].

Dietary strategies can also be developed to target native mucus degraders in cats and dogs. For example, prebiotic administration helps increase the levels of *Akkermansia* likely due to an increase in the number of goblet cells and mucus layer thickness [20]. Although the overall effect of prebiotics on mucus foragers remains to be investigated in cats and dogs, it was recently shown that cherry powder (high in prebiotics and polyphenolics) led to a five-fold difference in relative abundance of *Akkermansia* in feces of obese db/db mice compared to both lean and obese controls [183]. It is likely that at least some prebiotics strengthen gut health through an indirect effect on mucus microbial degraders in cats and dogs [199]. Other dietary alternatives to boost local mucus degraders include the use of polyphenols [200]. Finally, the growing conditions of *Akkermansia* and other microbes can be modified to obtain a different phenotype [201], but few data exist regarding the biochemical composition of intestinal mucus in cats and dogs to hypothesize what culture condition may lead to potentially advantageous down- or up-regulation of specific genes.

Another important line of research worth investigating is whether the presence of *Akkermansia* spp. and other mucus degraders in the mucus correlate with its abundance in luminal contents and feces, or if there is variation in the mucus adhesion properties as to open the possibility that the shed bacteria are different from those that are fixed into the mucosal layer. This may explain the apparent low concentration of *Akkermansia* in feces of cats and dogs because these bacteria may be closely attached to the mucosa, thus reducing its loss in feces. Interestingly, with other microbes such as in some strains of *Lactobacillus*, there is great variation in mucin adhesion properties and structures [202]. Therefore, it is feasible to hypothesize that each *Akkermansia* species or strain displays variation in their behavior in vivo, a possibility that has been proven in silico [67]. Feces of cats and dogs may contain a high degree of intestinal mucus as shown by studies in humans [40]. In this regard, it was reported that differences between lumen and mucosal communities may be quite small as a consequence of extensive mucus shedding and mixing in the lumen [175]. This is particularly important in light of new evidence suggesting the presence of potentially undiscovered *A. muciniphila* (see above).

Finally, it has been suggested that bacteria that live in the mucosa do not compete with the microbiota present in the lumen and therefore do not depend on dietary nutrients deriving from host food consumption [23,24]. However, the biochemical properties of mucin depend on the diet [203,204] and the glycan repertoire can select for distinct mucosa-associated bacteria [205]. Therefore, diet is also strongly, albeit indirectly, associated with the microbes attached to the mucosa. Moreover, it is unlikely that bacteria that are shed into the lumen cease all activities and die. In fact, ingested and salivary microbes may integrate into the native microbiome, although the alterations in the large intestine are mostly of limited extent [206,207].

## 7. Conclusions

In summary, microbial degradation of mucus is important for GI health and disease of cats and dogs, but this topic has received very little attention. *Akkermansia* is apparently not that universal across the animal kingdom, and thus far, there is no indication to believe that this taxon is prevalent or highly abundant in the digestive tract of cats and dogs. Other bacteria that have been detected in intestinal mucosa, such as members of Bacteroidaceae, Prevotellaceae, Clostridiales, *Faecalibacterium*, and the poorly studied Fusobacteria phylum, may deserve more attention as possible contributors to mucus degradation in the gut of cats and dogs. Remaining challenges include more research into the identities of mucus foragers in these companion animals, the search for dietary alternatives to boost these native mucus degraders, and the fate of mucosal bacteria shed into the lumen.

## Figures and Tables

**Table 1:** Summary of studies on the feline and canine gut microbiota based on molecular methods ^1^.

Animal Species	Origin, Number and Characteristics of Samples	Comments	Reference
Cats	Contents from stomach, duodenum, jejunum, ileum, and colon. n = 4 healthy, n = 1 healthy specific pathogen-free	Clone libraries. Verrucomicrobia was not detected, may be related to sequencing depth	[106]
Dogs	Contents from duodenum, jejunum, ileum, and colon. n = 6 healthy	Clone libraries. Verrucomicrobia was not detected, may be related to sequencing depth	[107]
Dogs	Duodenal brush cytology samples. n = 9 healthy, n = 10 with IBD	Clone libraries. Verrucomicrobia was not detected, may be related to sequencing depth	[108]
Dogs	Fecal samples. n = 8 healthy, n = 9 with chronic diarrhea	DGGE and FISH. Verrucomicrobia was not detected, likely related to methods	[109]
Dogs	Duodenal biopsies. n = 7 healthy, n = 7 with IBD	Clone libraries. Verrucomicrobia accounted for 0.1% of all reads	[110]
Cats and dogs	Fecal samples. n = 12 healthy dogs, n = 12 healthy cats	454-pyrosequencing. *Akkermansia* was only detected in one feline sample in low proportion (0.01%), may be related to sequencing depth	[111]
Dogs	Oral cavity. Subgingival plaque from n = 20 (10 kenneled dogs, study 1) and n = 31 (study 2)	Clone libraries. Verrucomicrobia was not detected, may be related to sequencing depth	[112]
Dogs	Fecal samples. n = 6 healthy at two time points	454-pyrosequencing. Verrucomicrobia was not detected, may be related to sequencing depth	[94]
Dogs	Gastric biopsies. n = 8 healthy	454-pyrosequencing. Verrucomicrobia was not detected, may be related to sequencing depth	[104]
Dogs	Biopsies from small intestinal mucosa. n = 6 healthy, n = 7 with moderate IBD, n = 7 with severe IBD	454-pyrosequencing. Verrucomicrobia was not detected, may be related to sequencing depth	[41]
Dogs	Fecal samples. n = 32 healthy, n = 12 with acute non-hemorrhagic diarrhea, n = 13 with acute hemorrhagic diarrhea, n = 9 with active IBD, n = 10 with therapeutically controlled idiopathic IBD	454-pyrosequencing. Verrucomicrobia was not detected, may be related to sequencing depth	[113]
Dogs	Fecal samples. n = 11 healthy	454-pyrosequencing. Verrucomicrobia was not detected, may be related to sequencing depth	[114]
Dogs	Fecal samples. n = 21 lean dogs, n = 22 obese dogs	454-pyrosequencing. Verrucomicrobia was detected only in one lean dog in low proportion (<0.01%), may be related to sequencing depth	[92]
Cats	Oral cavity. n = 11 healthy	Illumina MiSeq. Five uncultured types of Verrucomicrobia accounted for 0.01% of all reads	[115]
Cats	Fecal samples. n=30 healthy kittens at different time points	HiSeq Illumina. Verrucomicrobia was not detected, likely not related to sequencing depth	[95]
Cats	Oral cavity. n = 10 periodontally healthy, n = 10 with periodontitis	Clone libraries. Verrucomicrobia was not detected, may be related to sequencing depth	[116]
Dogs	Fecal samples. n = 13 healthy, n = 13 with acute diarrhea	454-pyrosequencing. Verrucomicrobia was not detected, may be related to sequencing depth	[117]
Cats	Oral cavity. Subgingival plaque bacterial communities. n = 20 with healthy gingiva, n = 50 with gingivitis, n = 22 with mild periodontitis	454-pyrosequencing. Verrucomicrobia was not detected, may be related to sequencing depth	[118]
Dogs	Fecal samples. n = 10 healthy, n = 12 with IBD	454-pyrosequencing. Verrucomicrobia was not detected, may be related to sequencing depth	[119]
Dogs	Subgingival plaque samples. n = 52 healthy at different time points	454-pyrosequencing. Verrucomicrobia was not detected, may be related to sequencing depth	[120]
Dogs	Jejunum. n = 8 with jejunal fistula at different time points	454-pyrosequencing. Verrucomicrobia was not detected, may be related to sequencing depth	[121]
Dogs	Fecal samples. n = 20 healthy	MiSeq Illumina. Verrucomicrobia was not detected, likely not related to sequencing depth	[122]
Cats and dogs	Fecal samples. n = 12 cats, n = 12 dogs healthy	454-pyrosequencing, Verrucomicrobia was not detected, may be related to sequencing depth	[123]
Dogs	Fecal samples. n = 30 puppies and some of their mothers (n = 16)	454-pyrosequencing. Verrucomicrobia was not detected, may be related to sequencing depth	[124]
Dogs	Fecal samples. n = 11 healthy, client-owned	MiSeq Illumina. Verrucomicrobia was not detected, likely not related to sequencing depth	[99]
Dogs	Fecal samples. n = 20 healthy, n = 20 diagnosed with meningoencephalomyelitis of unknown origin	MiSeq Illumina. Verrucomicrobia was not detected, likely not related to sequencing depth	[103]
Dogs	Fecal samples. n = 6 fed a natural diet, n = 5 fed a commercial diet	Verrucomicrobia was not detected, likely not related to sequencing depth	[125]
Dogs	Fecal samples. n = 8 healthy	MiSeq Illumina. Verrucomicrobia was not detected, likely not related to sequencing depth	[126]
Dogs	Fecal samples. n = 17 healthy, n = 27 overweight, n = 22 obese	Verrucomicrobia accounted for <0.001% of all reads	[93]
Dogs	Fecal and mucosa-associated. n = 13 healthy, n = 10 with colorectal epithelial tumors	MiSeq Illumina. Verrucomicrobia was not detected, likely not related to sequencing depth	[127]
Dogs	Duodenal and colonic biopsies. n = 9 with IBD, n = 15 with food-responsive diarrhea	MiSeq Illumina. Verrucomicrobia was not detected, likely not related to sequencing depth	[91]
Dogs	Fecal samples. n = 20 healthy, one additional dog with protein-losing enteropathy	MiSeq Illumina. Verrucomicrobia was not detected, likely not related to sequencing depth	[128]
Dogs	Fecal samples. n = 6 healthy	MiSeq Illumina. Verrucomicrobia was not detected, likely not related to sequencing depth	[129]
Cats	Fecal samples. n = 6, healthy	Verrucomicrobia was not detected, likely not related to sequencing depth	[130]
Dogs	Fecal samples. n = 27 fed a natural diet, n = 19 fed a commercial food	MiSeq Illumina. Verrucomicrobia was not detected, likely not related to sequencing depth	[131]
Dogs	Fecal samples. n = 168 healthy	454-pyrosequencing. Verrucomicrobia was not detected, may be related to sequencing depth	[96]
Dogs	Fecal samples. n = 25 with acute hemorrhagic diarrhea syndrome	Quantitative PCR assays. No members of Verrucomicrobia were searched for	[132]
Dogs	Fecal samples. n = 169 healthy	Verrucomicrobia was detected in low abundance (0.02–0.03%)	[101]
Dogs	Fecal samples. n = 27 weaned puppies, n = 74 unweaned puppies	MiSeq Illumina. Verrucomicrobia was not detected, likely not related to sequencing depth	[133]
Dogs	Fecal samples. n = 34 healthy, n = 15 with chronic enteropathy, n = 36 with exocrine pancreatic insufficiency	Quantitative PCR assays. No members of Verrucomicrobia were searched for	[134]
Dogs	Fecal samples. n = 24 healthy, n = 10 with food-responsive chronic enteropathy	MiSeq Illumina. Verrucomicrobia was not detected, likely not related to sequencing depth	[135]
Dogs	Colon biopsies. n = 22 with chronic inflammatory enteropathy, n = 11 healthy	FISH. *Akkermansia* was detected in the surface and the crypts. A higher abundance was detected in healthy dogs	[136]
Dogs	Fecal samples. n = 10 healthy, n = 10 with clinical diagnosis of diabetes mellitus	MiSeq Illumina. Verrucomicrobia was not detected, likely not related to sequencing depth	[137]
Cats and dogs	Fecal samples. n = 192 dogs, n = 46 cats	Verrucomicrobia, including *A. muciniphila* representatives, was 0.11% in cats and 0.02% in dogs	[138]
Dogs	Fecal samples. n = 10 healthy, n = 21 displaying conspecific aggressive behavior	MiSeq Illumina. Verrucomicrobia was not detected, likely not related to sequencing depth	[105]
Dogs	Fecal samples. n = 16 healthy	MiSeq Illumina. Verrucomicrobia was not detected, likely not related to sequencing depth	[139]
Dogs	Fecal samples. n = 49 healthy, n = 73 with chronic enteropathy	MiSeq Illumina. Verrucomicrobia was not detected, likely not related to sequencing depth	[140]
Dogs	Fecal samples. n = 8 healthy	MiSeq Illumina. Verrucomicrobia was not detected, likely not related to sequencing depth	[141]
Dogs	Fecal samples. n = 4 healthy, n = 4 with canine parvovirus	MiSeq Illumina. Verrucomicrobia was not detected, likely not related to sequencing depth	[142]
Dogs	Fecal samples. n = 8 healthy, n = 12 with food-responsive enteropathy	MiSeq Illumina. Verrucomicrobia was not detected, likely not related to sequencing depth	[143]
Dogs	Fecal samples. n = 76 with various clinical conditions	Verrucomicrobia was detected in low abundances ^2^	[144]
Cats	Fecal samples. n = 16 healthy	MiSeq Illumina. Verrucomicrobia was not detected, likely not related to sequencing depth	[145]
Dogs	Fecal samples. n = 90, half with and half without probiotics	PacBio RS II instrument. Verrucomicrobia was not detected, likely not related to sequencing depth	[146]

^1^ For studies using massive sequencing of marker genes (e.g., 16S rRNA gene), we did not attempt to look for Verrucomicrobia using the raw sequencing data. Therefore, the statement “Verrucomicrobia was not detected” may in some cases imply that the presence of Verrucomicrobia was not reported. ^2^ This paper mentioned “big percentages decreases” in the text but this is very difficult to visualize in their Figure 3A. In our experience, 454-pyrosequencing produces about one tenth the number of sequences currently produced with other technologies, for example the Illumina platforms [77]. IBD: Inflammatory Bowel Disease. DGGE: Denaturing Gradient Gel Electrophoresis. FISH: Fluorescent in situ hybridization.
